# Neighborhood Disadvantage, Built Environment, and Breast Cancer Outcomes: Disparities in Tumor Aggressiveness and Survival

**DOI:** 10.3390/cancers17091502

**Published:** 2025-04-29

**Authors:** Jie Shen, Yufan Guan, Supraja Gururaj, Kai Zhang, Qian Song, Xin Liu, Harry D. Bear, Bernard F. Fuemmeler, Roger T. Anderson, Hua Zhao

**Affiliations:** 1 Department of Public Health Sciences, School of Medicine, University of Virginia, Charlottesville, VA 22903, USA; 2 Department of Population and Community Health, College of Public Health, The University of North Texas Health Science Center at Fort Worth, Fort Worth, TX 76107, USA; 3 Department of Gerontology, Donna M and Robert J Manning College of Nursing and Health Sciences, University of Massachusetts, Boston, MA 02125, USA; 4 School of Public Health and Emergency Management, Southern University of Science and Technology, Shenzhen 518055, China; 5 Departments of Surgery, School of Medicine, Virginia Commonwealth University, Richmond, VA 23284, USA; 6 Departments of Family Medicine, School of Medicine, Virginia Commonwealth University, Richmond, VA 23284, USA

**Keywords:** allostatic load, breast cancer aggressiveness, survival, area deprivation index, built environment

## Abstract

Background: Breast cancer disparities persist globally, with growing evidence implicating neighborhood and built environmental factors in disease outcomes. Methods: This study investigates the associations between neighborhood disadvantage, environmental exposures, and breast tumor characteristics and survival among 3041 stage I–III breast cancer patients treated at the University of Virginia Comprehensive Cancer Center (2014–2024). Neighborhood disadvantage was assessed via the Area Deprivation Index (ADI), while environmental exposures included PM2.5, green space (NDVI), and food indices (modified retail food environment index (mRFEI), retail food activity index (RFAI)). Multivariable regression and Cox models adjusted for demographic, socioeconomic, and clinical covariates were employed. Results: A higher ADI score was associated with aggressive tumor characteristics, including advanced stage (Odds Ratio (OR) = 1.06, 95% Confidence Interval (CI):1.01–1.11), poor differentiation (OR = 1.07, 1.01–1.15), ER-negative status (OR = 1.06, 1.01–1.12), and triple-negative breast cancer (TNBC) (OR = 1.08, 1.02–1.16), as well as younger diagnosis age (β = −0.22, −0.36 to −0.09). PM2.5 exposure was correlated with advanced tumor stage (OR = 1.24, 1.09–1.40 for stage III) but paradoxically predicted improved survival (Hazard Ratio (HR) = 0.71, 0.63–0.82). The food environment indices showed subtype-specific survival benefits: higher mRFEI and RFAI scores were linked to reduced mortality in ER-negative (HR = 0.45, 0.23–0.85 and HR = 0.61, 0.38–0.97) and TNBC (HR = 0.40, 0.18–0.90 and HR = 0.48, 0.26–0.87) patients. NDVI scores exhibited no significant associations. Conclusion: Our findings underscore the dual role of neighborhood disadvantage and the built environmental in breast cancer outcomes. While neighborhood disadvantage and PM2.5 exposure elevate tumor aggressiveness, survival disparities may be mediated by other factors. Improved food environments may enhance survival in aggressive subtypes, highlighting the need for integrated interventions addressing socioeconomic inequities, environmental risks, and nutritional support needs.

## Simple Summary

This study examined how neighborhood disadvantage and environmental exposures influenced breast cancer characteristics and survival among 3041 patients at the University of Virginia Comprehensive Cancer Center. Higher neighborhood disadvantage and PM2.5 exposure were associated with more aggressive tumor features, although PM2.5 unexpectedly predicted improved survival. Healthier food environments were linked to better survival rates for aggressive subtypes like ER-negative and triple-negative breast cancer, highlighting the need for integrated approaches that address environmental and socioeconomic factors.

## 1. Introduction

Breast cancer remains the most diagnosed malignancy among women worldwide, with profound implications for morbidity, mortality, and quality of life [1]. While advancements in early detection and treatment have improved outcomes, significant disparities persist in disease incidence, tumor biology, and survival across populations [2,3]. Traditional research has focused extensively on individual-level risk factors, such as genetic predisposition, reproductive history, and lifestyle behaviors [4]. However, evidence underscores the critical role of neighborhood and built environmental factors in shaping cancer risk, progression, and survival [5,6]. The neighborhood and built environment—comprising factors such as residential segregation, socioeconomic status (SES), access to healthcare, availability of healthy food, walkability, green spaces, and exposure to environmental pollutants—have been increasingly recognized as potential contributors to cancer disparities [5,6,7,8]. 

Emerging research has demonstrated that neighborhood-level SES is associated with variations in breast cancer incidence, tumor aggressiveness, and survival. For instance, individuals residing in lower SES neighborhoods often have more aggressive tumor subtypes and poorer survival outcomes [9,10,11,12,13,14,15]. The built environment may also play a role in breast cancer outcomes [16,17]. Diet and physical activity are established modifiable risk factors for breast cancer, with higher adherence to healthy lifestyle behaviors being linked to better prognoses [18]. Conversely, exposure to fine particulate matter (PM2.5) and endocrine-disrupting chemicals in urban settings has been associated with increased breast cancer risk and more aggressive tumor characteristics [19,20,21,22]. Despite these insights, there remains a gap in understanding the complex interplay between neighborhood characteristics, built environment factors, and breast cancer outcomes. A comprehensive examination of these relationships could provide crucial insights into potential interventions aimed at mitigating disparities and improving breast cancer outcomes.

This manuscript aims to explore the associations that neighborhood and built environment factors have with breast tumor characteristics and survival. Utilizing valuable breast cancer patients seen at the University of Virginia Comprehensive Cancer Center, we attempted to analyze (1) the associations between neighborhood disadvantage, represented as the Area Deprivation Index (ADI), and breast tumor characteristics at the time of diagnosis (e.g., tumor stage, grade, ER status, and TNBC status), age at cancer diagnosis, and overall survival; and (2) the association of selected built environment factors, including PM2.5 levels, the normalized difference vegetation index (NDVI), the modified food environmental index (mRFEI), and the retail food activity index (RFAI), with breast tumor characteristics and overall survival. By integrating epidemiological, environmental, and socio-behavioral perspectives, this study seeks to contribute to the growing body of literature addressing the impact of contextual factors on breast cancer prognosis and health equity.

## 2. Materials and Methods

### 2.1. Study Population

In this study, 3041 female breast cancer patients were identified from the breast cancer patient pool seen at the University of Virginia Comprehensive Cancer Center in the last decade (2014 to 2024). The selection criteria included (1) newly diagnosed stage I to III breast cancer, (2) no prior cancer diagnosis except non-melanoma skin cancer, and (3) living in the catchment area of the University of Virginia Comprehensive Cancer Center, which includes 3.2 million residents from a large area that includes 87 counties throughout northern, central, southside, and southwestern Virginia, as well as eastern West Virginia. The University of Virginia institutional review board approved this study.

### 2.2. Area Deprivation Index (ADI)

In this study, we assessed neighborhood-level socioeconomic disadvantage using the Area Deprivation Index (ADI) developed by the Neighborhood Atlas [23]. The 2022 version of the ADI was derived from the 2014–2018 American Community Survey 5-year estimates provided by the U.S. Census Bureau. The ADI assigns rankings to neighborhoods based on a composite measure of factors related to income, education, employment, and housing quality. This study used the national percentile rankings of ADI scores, which range from 1 to 100, with higher percentiles indicating greater socioeconomic disadvantage.

### 2.3. Built Environment Factors

PM2.5: The data for PM2.5 were gathered using published satellite-derived datasets that combine information from satellites and ground-based measurements of PM2.5 by statistically enhancing geographically based estimates within a geographically weighted regression (GWR) framework [24]. More details on the exposure assessment are described elsewhere [24]. Participants’ residential addresses at each exam point were geocoded and linked to the estimated PM2.5 concentrations. The cumulative-term PM2.5 exposure (μg/m^3^) was calculated as the average concentration over one-, three-, and five-year periods preceding each participant’s breast cancer diagnosis, based on their geocoded residential location at the time of diagnosis. For the primary analysis, we focused on the five-year average PM2.5 exposure prior to diagnosis, with the one- and three-year averages included in sensitivity analyses (Supplementary Tables S1 and S2).

**Normalized difference vegetation index (NDVI):** Surrounding greenness was assessed using the normalized difference vegetation index (NDVI), a satellite-derived measure that reflects overall land surface greenness but does not differentiate between vegetation types. NDVI values were obtained from the Global Inventory Modeling and Mapping Studies (GIMMS) and the Moderate Resolution Imaging Spectroradiometer (MODIS) datasets. All the NDVI data used in this study were downloaded from NASA’s Earthdata Search Portal (https://search.earthdata.nasa.gov/search, accessed on 16 March 2025). In this study, the 1-km buffer was available 1, 2, 3, and 5-year prior to the cancer diagnosis. We used NDVI 5-year as our primary measure of interest in main analyses, and other years as measures in a sensitivity analysis for comparison purposes.

**Modified retail food environment index (mRFEI):** The mRFEI is a metric developed by the Centers for Disease Control and Prevention (CDC) to assess the availability of healthy food retailers within a specific area. A higher mRFEI score indicates a greater proportion of healthy food retailers in the area. It was measured at the census tract level as a percentage of healthy food retailers in all qualified food. In this study, we obtained this from the data at the census track level from the Centers for Disease Control and Prevention’s (CDC’s) Division of Nutrition, Physical Activity, and Obesity.

**The retail food activity index (RFAI):** The RFAI is a novel metric designed to assess the proportion of consumer visits to healthy food retailers within a specific area, such as a census tract [25]. Unlike traditional indices that focus solely on the geographic distribution of food outlets, the RFAI incorporates actual consumer behavior by analyzing visit patterns to these retailers. This approach provides a more dynamic understanding of the food environment.

### 2.4. Tumor Characteristics and Overall Survival

The tumor characteristics included tumor stage, tumor grade, tumor subtype, and age at cancer diagnosis. The tumor stage was classified as I, II, or III. The tumor grade was categorized as well-differentiated or poorly differentiated. The tumor subtype included ER and TNBC status. The overall survival was determined as the time from the primary diagnosis to the point of death from any cause. Death was determined by the Cancer Registry at the University of Virginia Comprehensive Cancer Center and treated as a dichotomous variable. Censoring was calculated using the date of death (with cause of death) or last known follow-up.

### 2.5. Covariates

The key characteristics included demographics (e.g., age and race), menopausal status, individual SES factors (e.g., employment, marital status, insurance type), lifestyle factors (e.g., tobacco use and alcohol consumption), and cancer treatment (e.g., chemotherapy and radiation). Menopausal status was classified into three categories: ‘Premenopausal’, ‘Postmenopausal’, and ‘Other’, with the ‘Other’ category including individuals who had undergone ablation, hysterectomy, or other procedures affecting menopausal status. Marital status was categorized as ‘Married’ and ‘Other’, with the ‘Other’ category including individuals who were divorced, legally separated, single, widowed, in a significant relationship, or classified under ‘Other’. Insurance type was classified into five categories: ‘Self-pay’, ‘Private’, ‘Medicaid’, ‘Medicare’, and ‘Other’. Alcohol consumption and tobacco use were both categorized as ‘Ever’ and ‘Never’ to distinguish individuals with a history of use from those who had never engaged in these behaviors. The clinical treatment variables included chemotherapy and radiation treatment, each categorized as ‘Yes’ or ‘No’ to indicate whether individuals had received these treatments.

### 2.6. Statistical Analysis

Descriptive statistics were applied to each demographic, lifestyle, and SES factor as well as cancer treatment to summarize the distribution of key characteristics across the study population. The same procedure was used to analyze the neighborhood variables, including ADI, PM2.5 levels, the NDVI score, and the food environment index scores (mRFEI and RFAI). To assess the relationship of the ADI score and built environment factors with the tumor stage and grade, a multinomial logistic regression model was employed. Similarly, a logistic regression model was used to assess the relationships of the ADI score and built environment factors with ER and TNBC status. Key covariates, including age, race, employment status, marital status, insurance type, menopausal status, tobacco use, and alcohol consumption, were included in the models. To assess the relationship of the ADI score and built environment factors with the age at cancer diagnosis, a linear regression analysis was applied. Covariates were included in the analysis as appropriate. To further examine the relationship between the ADI score and built environment factors and breast cancer mortality, the Cox Proportional Hazards regression model was employed. A series of models were constructed with sequential adjustments for covariates to refine the analysis. Model 2 was adjusted for demographic variables (age, race, and menopausal status). Model 3 incorporated demographic variables and socioeconomic status (employment, marital status, and insurance type). Model 4 included demographic variables and lifestyle factors (tobacco use and alcohol consumption). Model 5 was adjusted for demographic variables and clinical factors, including tumor stage, subtype, and treatment. Finally, Model 6 was fully adjusted to account for all the covariates. Given a total of 3041 breast cancer patients and 494 deaths during the follow-up, we had 71% of power to detect an HR of 1.05 for ADI score, and 87% of power to detect an HR of 1.15 for PM2.5. A stratified analysis was subsequently conducted to explore the association of ADI score and built environment factors with breast cancer mortality, stratified by tumor subtype. Additionally, a sensitivity analysis was performed for PM2.5 to evaluate the robustness of the findings. All the analyses were conducted using R, version 3.5.2 (R Foundation for Statistical Computing). All the statistical tests were 2-sided, and statistical significance was assessed at α < 0.05.

## 3. Results

Table 1 describes the study population. Overall, the study included a total of 3041 breast cancer patients with a mean age of 65.71 years. The majority of the study population was White (82.47%) and about half of the participants were postmenopausal (50.08%). In terms of socioeconomic factors, 31.70% of the participants were employed, while 41.80% were retired. The majority (58.07%) were married. Over 80% of the participants had insurance, including private insurance, Medicare, Medicaid, etc. Lifestyle factors varied among participants, with 55.80% and 41.04% reporting a history of alcohol consumption and tobacco use. Most patients were diagnosed at an early tumor stage, with 60.37% classified as stage I, followed by 27.43% at stage II, and 11.61% at stage III. Among those with available information, 22.85% had moderately differentiated tumors, 13.75% had poorly differentiated tumors, and 10.82% had well-differentiated tumors. For hormone receptor status, 64.12% of the tumors were estrogen receptor (ER)-positive, whereas 11.87% were ER-negative and 6.94% were TNBC. In terms of cancer treatment, 35.81% and 51.96% of the patients had received chemotherapy and radiation therapy. The mean ADI score at the state level was 53.4. The mean PM2.5 exposure level was 7.13 µg/m^3^. The mean NDVI score was 0.61, while the mean mRFEI score was 3.77. Additionally, the RFAI score had a mean value of 17.43.

Table 2 presents the associations of the ADI score and built environment variables with the breast tumor characteristics. The results are adjusted for key demographic (e.g., age, race, and menopausal status), socioeconomic (e.g., employment, marital status, insurance), and lifestyle (e.g., tobacco use, and alcohol use) factors. Higher ADI scores (as a continuous variable) were significantly associated with more aggressive tumor characteristics. Specifically, higher ADI scores were associated with a 6% increased likelihood of having stage III (OR = 1.06, 95% CI: 1.01–1.11) and a 7% increased likelihood of poorly differentiated (OR = 1.07, 95% CI: 1.01–1.15) tumors. The ADI score was also positively associated with ER− status (OR = 1.06, 95% CI: 1.01–1.12) and TNBC (OR = 1.08, 95% CI: 1.02–1.16). When the ADI score was categorized into low (1–50) vs. high (51–100), patients in more disadvantaged neighborhoods had 1.58 times higher odds of poorly differentiated tumors (95% CI: 1.14–2.17), a 1.31-fold higher odds of ER− tumors (95% CI: 1.03–1.66), and a 1.51-fold higher odds of TNBC (95% CI: 1.12–2.05) compared to those in less disadvantaged neighborhoods. Higher PM2.5 levels were associated with an increased risk of advanced tumor stage. Each unit increase in PM2.5 exposure was associated with 23% increased odds of stage II tumors (OR = 1.23, 95% CI: 1.12–1.34) and 24% increased odds of stage III tumors (OR = 1.24, 95% CI: 1.09–1.40). When categorized into high vs. low PM2.5 exposure, patients in higher exposure areas had 56% increased odds of stage II tumors (OR = 1.56, 95% CI: 1.31–1.86) and a 43% increased odds of stage III tumors (OR = 1.43, 95% CI: 1.11–1.84). No significant association was observed between NDVI levels and any of the breast tumor characteristics. In terms of the food environment, the mRFEI, which measures access to healthy food options, showed an association with tumor differentiation. Individuals residing in areas with higher mRFEI scores had 1.59 times higher odds of having moderately vs. well-differentiated tumors (95% CI: 1.14–2.23). No significant associations were observed for tumor stage, receptor status, or TNBC. The RFAI score was not significantly associated with the tumor characteristics, although a borderline positive association was observed for ER− breast tumors (OR = 1.27, 95% CI: 0.99–1.63).

Table 3 presents the associations of the ADI score and built environment variables with the age at breast cancer diagnosis, both overall and stratified by ER status and TNBC status. Higher ADI scores were significantly associated with a younger age at breast cancer diagnosis in the overall sample (β = −0.22, 95% CI: −0.36 to −0.09), indicating that individuals residing in more socioeconomically disadvantaged neighborhoods were diagnosed at earlier ages. This association remained significant among ER+ cases (β = −0.27, 95% CI: −0.43 to −0.10) and non-TNBC cases (β = −0.23, 95% CI: −0.38 to −0.08). However, no significant association was observed for ER− or TNBC cases. Higher PM2.5 levels were associated with a slightly older age at breast cancer diagnosis in the overall sample (β = 0.33, 95% CI: 0.01 to 0.65), although this association was not statistically significant in the stratified analyses for ER status or TNBC. Higher NDVI levels were not significantly associated with age at diagnosis across all breast cancer subtypes. The mRFEI score showed a weak inverse association with age at diagnosis (β = −0.64, 95% CI: −1.36 to 0.07), but none of the findings reached statistical significance. Conversely, the RFAI score was significantly associated with a younger age at breast cancer diagnosis. Women in neighborhoods with higher RFAI scores were diagnosed at an earlier age in the overall sample (β = −0.94, 95% CI: −1.55 to −0.32) and among ER-positive cases (β = −0.84, 95% CI: −1.59 to −0.08). Although not statistically significant, a similar trend was observed among ER-negative (β = −1.09, 95% CI: −3.17 to 0.99) and TNBC (β = −2.49, 95% CI: −5.30 to 0.32) cases.

The mean (SD) follow-up time for the study participants was 31.2 (19.6) months overall. The mean (SD) follow-up time for patients who were alive was 60.7 (38.6) months and 31.2 (18.4) months for patients who died.

Table 4 presents the associations of the ADI score and built environment variables with overall survival among the breast cancer patients across the six models with increasing levels of adjustment. In Model 1 (crude) and Model 2 (adjusted demographics), higher ADI scores were associated with a significant increase in mortality risk (HR = 1.06, 95% CI: 1.02–1.10 and HR = 1.04, 95% CI: 1.01–1.09, respectively). However, after further adjusting for socioeconomic, lifestyle, and clinical factors (from Models 3 to 6), this association was no longer significant. A surprising inverse association was observed between PM2.5 exposure and mortality risk. In the fully adjusted model (Model 6), higher PM2.5 levels were associated with a 29% lower hazard of mortality (HR = 0.71, 95% CI: 0.63–0.82). This trend was consistent across all the models, with the effect strengthening after adjusting for individual SES, lifestyle, and clinical characteristics. Higher NDVI levels showed no significant association with overall survival. Neither the mRFEI nor the RFAI score was significantly associated with overall survival. However, a marginal association was noted between the mRFEI score and overall survival in the fully adjusted model (Model 6) (HR = 0.79, 95% CI: 0.61–1.03).

Table 5 presents the associations of the ADI score and built environment factors with overall survival stratified by ER and TNBC status. The ADI score was not significantly associated with survival in any of the tumor subtypes. Higher PM2.5 levels were consistently associated with lower mortality risk across all the tumor subtypes, with the strongest inverse association observed among TNBC cases (HR = 0.57, 95% CI: 0.39–0.83). Higher NDVI levels were not significantly associated with survival across the tumor subtypes. The mRFEI score was associated with improved survival in ER− (HR = 0.45, 95% CI: 0.23–0.85) and TNBC (HR = 0.40, 95% CI: 0.18–0.90) cases, but not in ER-positive or non-TNBC cases. Similarly, the RFAI score showed a significant inverse association with mortality risk in ER-negative (HR = 0.61, 95% CI: 0.38–0.97) and TNBC (HR = 0.48, 95% CI: 0.26–0.87) cases, but not in ER-positive or non-TNBC cases.

## 4. Discussion

This study examined the associations of neighborhood deprivation, represented by ADI scores, and built environment factors, including PM2.5, NVDI, mRFEI, and RFAI, with breast tumor characteristics, age at diagnosis, and overall survival, using data from 3041 stage I to III female breast cancer patients newly diagnosed in the last 10 years at the University of Virginia Comprehensive Cancer Center. In this study, we found that a higher ADI score was associated with more aggressive tumor characteristics and younger age of diagnosis, but its direct impact on overall survival was attenuated after adjusting for individual SES, lifestyle, and clinical factors. We also observed that higher PM2.5 exposure was associated with advanced tumor stages but paradoxically linked to improved overall survival. In addition, both higher mRFEI and RFAI scores were associated with longer overall survival among ER− and TNBC patients.

Our findings on the association between ADI scores and aggressive breast tumor characteristics is consistent with several previous studies [9,10,11,12,13,14,15]. For example, in a study of 5027 breast cancer patients, Goel et al. found that patients living in the most disadvantaged neighborhoods were more likely to have TNBC and a higher tumor stage and grade, compared with those in the most advantaged neighborhoods [12]. In another study of 36,795 breast cancer patients, compared to women in the lowest quintile of NDI, those living in the highest quintile were more likely to have TNBC [15].

In addition, both studies reported that higher neighborhood deprivation was associated with an increased hazard of breast cancer mortality even after adjusting for various types of covariates [12,15]. We observed similar risk association in the crude model and the model adjusted for demographics alone. However, after adjusting for additional SES, lifestyle, and clinical factors, the significant association was no longer present. This may suggest that a certain degree of risk association between ADI scores and breast cancer mortality can be modified or mediated by SES, lifestyle, and clinical factors. It may also reflect the difference between our study and theirs. Compared to those two studies, our study has smaller sample size (3041 vs. 5027 and 36,795) and a shorter follow-up time (31.2 months year vs. more than 60 months in both studies). The study populations in both studies are more racially and SES diverse. Also, one of the studies included stage IV breast cancer patients [12]. All those factors may contribute to the discrepancy between our study and theirs. Intriguingly, in the study by Barber et al. [15], the significant relationship between neighborhood deprivation and increased breast cancer mortality was only observed among non-Hispanic White women, but not among non-Hispanic Black women, regardless of the modifying neighborhood characteristics considered. Similar racial differences was also observed in our study (White women (HR = 1.06, 95% CI: 1.01, 1.10) and Black women (HR = 0.96, 0.87, 1.08), after adjusting for age and menopausal status).

An earlier age at breast cancer diagnosis is often linked to more aggressive tumor characteristics [26]. In this study, we found that a higher ADI score was associated with a younger age at diagnosis, and this association remained significant even after adjusting for tumor stage, grade, and subtype. These findings provide further evidence supporting the role of neighborhood disadvantage in breast tumor aggressiveness. Interestingly, the significant association was observed only among ER+ and non-TNBC patients. Given that higher ADI was also linked to ER− and TNBC tumors, our results suggest that neighborhood disadvantage broadly influences tumor aggressiveness. Women in more disadvantaged areas are not only more likely to develop aggressive ER− tumors and TNBC but also tend to be diagnosed at a younger age, even when their tumors are less aggressive. The underlying causes of the observed differences by tumor subtype are unclear. It may be attributed to the role of genetics in determining the age of diagnosis for ER− and TNBC tumors. In contrast, for ER+ and non-TNBC tumors, non-genetic factors, such as neighborhood disadvantage, may have a greater influence on the age at diagnosis.

The results on PM2.5 exposure are intriguing. On one hand, we found that higher PM2.5 exposure was associated with more advanced tumor stage at diagnosis. On the other hand, increasing PM2.5 levels were linked to improved overall survival across all tumor subtypes. The observed association with breast tumor aggressiveness aligns with the existing literature [26]. For example, research from the National Cancer Institute of Mexico indicated that long-term PM2.5 exposure was linked to more aggressive breast cancer phenotypes, including larger tumor sizes and a higher likelihood of TNBC [26]. However, many existing studies have reported a positive association between higher PM2.5 levels and shorter breast cancer survival [27,28,29]. A meta-analysis, for instance, demonstrated a 17% increase in breast cancer mortality for every 10 µg/m^3^ rise in PM2.5 levels [28]. Another study found that higher PM2.5 exposure was associated with decreased survival among breast cancer patients, particularly those diagnosed at earlier stages [29]. This discrepancy may be due to technical limitations in our study. Our follow-up period is relatively short (median: 31.2 months), making deaths more likely to occur among breast cancer patients with more advanced-stage disease. As shown by Hu et al., the detrimental effects of PM2.5 exposure on survival were more pronounced in individuals with early-stage cancers [29]. Thus, the full impact of PM2.5 on cancER-related mortality may not yet be fully realized in our cohort. Additionally, our study population is geographically limited compared to nationwide and statewide studies [27,28,29,30], resulting in a narrower PM2.5 exposure range and a greater susceptibility to unexpected bias and confounding factors. The unexpected survival benefit associated with higher PM2.5 levels may also be explained by urban–rural healthcare disparities. Higher PM2.5 concentrations are typically found in urban areas, which often have better medical infrastructure, advanced cancer treatments, and higher screening rates. This is particularly relevant to our study, as our cancer center’s catchment area includes a large proportion of rural regions with limited healthcare access. Thus, PM2.5 may be acting as a surrogate marker for urban versus rural residence. In this context, the association between greater PM2.5 exposure and improved survival may reflect underlying disparities in healthcare access rather than a direct protective effect of air pollution. While air pollution is known to promote inflammation and oxidative stress [31], our findings may suggest that improved healthcare accessibility in urban settings may mitigate these adverse effects on survival. Future studies should investigate the biological versus contextual mechanisms underlying this association, as well as potential interactions between air pollution exposure and healthcare quality.

Another interesting finding from our study is the beneficial effect of a better food environment, as measured by the mRFEI and RFAI, on breast cancer survival in ER− and TNBC cases. While the mRFEI—and to a lesser extent, the RFAI—have been used to study neighborhood food environments in relation to various health outcomes [25,32,33], there is limited research specifically examining their relationship with breast tumor characteristics and patient survival. The mRFEI assesses the proportion of healthy food outlets relative to less healthy options within a specific area. A higher mRFEI score indicates better access to nutritious foods. Poor food environments (low mRFEI score) are associated with higher consumption of processed, high-fat, and low-nutrient foods, which can contribute to obesity, inflammation, and insulin resistance—factors linked to more aggressive breast cancer subtypes such as ER− and TNBC [34]. Studies suggest that diets rich in fruits, vegetables, whole grains, and lean proteins are associated with better outcomes, especially in ER− and TNBC patients [35]. Also, a study analyzing ZIP code-level data found that areas with lower mRFEI scores had significantly higher breast cancer mortality rates [36]. The RFAI measures the density and accessibility of various food retailers within a community, reflecting the local food environment’s healthfulness. RFAI levels tend to be higher in urban areas than in rural regions. Higher RFAI levels in urban areas might correlate with improved breast cancer survival due to better medical infrastructure, access to specialized oncology care, and higher screening rates and early detection. Urban areas are also associated with higher rates of breast cancer screening and consequently earlier detection of disease. This may also help explain that higher RFAI levels were associated with a younger age of diagnosis among all women and specifically among ER+ cases. Additionally, research on urban food deserts—areas with limited access to healthy food options—found that breast cancer patients living in these areas had a 5-year survival rate of 78%, compared to 80% for those in non-food desert areas, suggesting that limited access to nutritious foods may negatively impact survival [37]. Given the limited direct evidence linking mRFEI and RFAI to breast tumor characteristics and survival, further research is warranted to explore this potential association. Understanding how the quality of the retail food environment influences breast cancer outcomes could inform public health interventions aimed at improving food environments and, consequently, cancer prognosis.

There are limitations to the present analysis. Although the ADI and built environment factors capture the multidimensionality of neighborhood information, their interpretations cannot be easily translated into an intervention. We measured ADI scores and built environment factors at a single time point, which may not reflect changes in the neighborhood, length of residency, changes in address, or cumulative exposure to neighborhood information over the course of a patient’s life. Second, varying degrees of data were missing on some key variables, such as tumor grade and ER status. Some cases were therefore excluded from the relevant analyses. Third, the follow-up period is short so the impact of the ADI score and built environment on breast cancer mortality may not yet be fully realized in our cohort. Fourth, the catchment area is narrow, which may limit the range of the exposure and consequently reduce the statistical power of our analysis. Fifth, because this study used medical record data, which have limited individual-level data, we were unable to adjust for some individual-level confounders, including diet and physical activity. In addition, our results may have limited generalizability to geographies beyond southwestern Virginia.

In summary, our study highlights the complex interplay between neighborhood disadvantage and environmental exposures in shaping breast cancer outcomes in a largely rural area of southwestern Virginia and eastern West Virginia. Higher ADI scores were associated with more aggressive tumor characteristics and shorter survival, though the relationship with survival was attenuated after adjusting for SES, lifestyle, and clinical variables. Although high PM2.5 levels were associated with higher tumor grade, they were linked to better overall survival rates, possibly driven by healthcare accessibility rather than direct environmental effects. In addition, food environment improvements may offer survival benefits for aggressive breast cancer subtypes, warranting further investigation into the role of nutrition in cancer survivorship. Additional studies with longer follow-up are needed to further clarify the results observed in this study. Also, studies to explore the biological link that ADI scores and the built environment have with breast tumor characteristics and survival is warranted. Finally, studies specific to rural or urban areas are needed to further explore the impact of geographic regions.

## Figures and Tables

**Table 1:** Demographic, clinical, and neighborhood characteristics of the study population.

Variables	
Age, Mean (SD)	N (%)
Race	65.71 (12.98)
White	2508 (82.47)
Black	347 (11.41)
Other	178 (5.85)
Missing	8 (0.26)
Menopausal Status	
Premenopausal	265 (8.71)
Postmenopausal	1523 (50.08)
Other *	878 (28.87)
Missing	375 (12.33)
Employment status	
Employed	964 (31.70)
Unemployment	372 (12.23)
Disabled	190 (6.25)
Retired	1271 (41.80)
Missing	244 (8.02)
Marital Status	
Married	1766 (58.07)
Others ^#^	1269 (41.73)
Missing	6 (0.20)
Insurance Type	
Yes	2494 (82.02)
No	433 (14.24)
Missing	117 (3.85)
Alcohol	
Never	1293 (42.52)
Ever	1697 (55.80)
Missing	51 (1.68)
Tobacco	
Never	1762 (57.94)
Ever	1, 248 (41.04)
Missing	31 (1.02)
Tumor Stage	
I	1836 (60.37)
II	834 (27.43)
III	353 (11.61)
Missing	18 (0.59)
Tumor Grade	
Well differentiated	329 (10.82)
Moderately differentiated	695 (22.85)
Poorly differentiated	418 (13.75)
Missing	1599 (52.58)
ER Status	
Negative	361 (11.87)
Positive	1950 (64.12)
Missing	730 (24.01)
Tri-negative	
Negative	2446 (80.43)
Positive	211 (6.94)
Missing	364 (11.97)
Chemotherapy	
Yes	1089 (35.81)
No	1952 (64.19)
Radiation	
Yes	1580 (51.96)
No	1461 (48.04)
Death	
Yes	494 (16.24)
No	2537 (83.43)
Missing	10 (0.33)
Median Follow-up Time (years)	31.2 (19.6)
ADI, Mean (SD)	53.4 (23.4)
PM2.5, Mean (SD)	7.13 (0.99)
NDVI, Mean (SD)	0.61 (0.07)
mRFEI, Mean (SD)	3.77 (8.06)
RFAI, Mean (SD)	

* Others included ablation, hysterectomy, and others. ^#^ Others included divorced, legally separated, single, widowed, and others.

**Table 2:** Associations of ADI score and built environment variables with tumor characteristics.

	Tumor Stage		Tumor Grade		ER Status	TNBC Status
	II vs. I	III vs. I	Moderately vs. Well	Poorly vs. Well	Negative vs. Positive	Yes vs. No
	OR (95% CI) *	OR (95% CI) *	OR (95% CI) *	OR (95% CI) *	OR (95% CI) *	OR (95% CI) *
ADI (Continuous)	1.03 (0.99, 1.07)	1.06 (1.01, 1.11)	1.02 (0.96, 1.08)	1.07 (1.01, 1.15)	1.06 (1.01, 1.12)	1.08 (1.02, 1.16)
ADI (Categorical)						
Low (1~50)	Reference	Reference	Reference	Reference	Reference	Reference
High (51~100)	1.16 (0.98, 1.39)	1.21 (0.94, 1.55)	1.20 (0.90, 1.60)	1.58 (1.14, 2.17)	1.31 (1.03, 1.66)	1.51 (1.12, 2.05)
PM2.5 (continuous)	1.23 (1.12, 1.34)	1.24 (1.09, 1.40)	1.01 (0.86, 1.17)	1.08 (0.91, 1.28)	1.03 (0.91, 1.17)	1.02 (0.87, 1.19)
PM2.5 (categorical)						
Low	Reference	Reference	Reference	Reference	Reference	Reference
High	1.56 (1.31, 1.86)	1.43 (1.11, 1.84)	1.08 (0.78, 1.50)	1.13 (0.77, 1.65)	1.07 (0.84, 1.37)	1.21 (0.89, 1.64)
NDVI (continuous)	1.62 (0.49, 5.41)	0.96 (0.20, 4.74)	0.25 (0.03, 2.02)	0.37 (0.04, 3.86)	3.37 (0.66, 18.79)	3.46 (0.45, 30.91)
NDVI (Categorical)						
Low	Reference	Reference	Reference	Reference	Reference	Reference
High	1.06 (0.89, 1.26)	0.95 (0.74, 1.21)	0.81 (0.61, 1.06)	0.79 (0.58, 1.09)	0.99 (0.79, 1.26)	0.97 (0.72, 1.30)
mRFEI						
Low (=0)	Reference	Reference	Reference	Reference	Reference	Reference
High (>0)	1.07 (0.87, 1.31)	1.26 (0.95, 1.65)	1.59 (1.14, 2.23)	1.16 (0.79, 1.71)	0.94 (0.71, 1.23)	1.19 (0.89, 1.61)
RFAI						
Low (=0)	Reference	Reference	Reference	Reference	Reference	Reference
High (>0)	1.07 (0.90, 1.28)	1.04 (0.82, 1.33)	1.04 (0.79, 1.37)	1.01 (0.74, 1.38)	1.27 (0.99, 1.63)	1.07 (0.79, 1.46)

* Adjusted with age, race, employment, marital status, insurance, menopausal status, tobacco use, and alcohol use.

**Table 3:** Association between ADI score and built environment variables and age at cancer diagnosis.

	Overall	ER+ (N = 1950)	ER− (N = 361)	TNBC (N = 211)	Non-TNBC (N = 2466)
	Coefficient (95% CI) *	Coefficient (95% CI) *	Coefficient (95% CI) *	Coefficient (95% CI) *	Coefficient (95% CI) *
ADI	−0.22 (−0.36, −0.09)	−0.27 (−0.43, −0.10)	0.04 (−0.40, 0.47)	0.11 (−0.52, 0.73)	−0.23 (−0.38, −0.08)
PM2.5	0.33 (0.01, 0.65)	0.30 (−0.10, 0.69)	−0.01 (−0.99, 0.99)	0.70 (−0.82, 2.22)	0.30 (−0.05, 0.65)
NDVI	2.29 (−1.88, 6.45)	3.57 (−1.38, 8.51)	3.73 (−10.90, 18.35)	−3.22 (−23.38, 16.95)	3.04 (−1.39, 7.47)
mRFEI					
Low (=0)	Reference	Reference	Reference	Reference	Reference
High (>0)	−0.64 (−1.36, 0.07)	−0.28 (−1.17, 0.60)	−0.87 (−3.17, 1.43)	−0.33 (−3.63, 2.98)	−0.49 (−1.27, 0.29)
RFAI					
Low (=0)	Reference	Reference	Reference	Reference	Reference
High (>0)	−0.94 (−1.55, −0.32)	−0.84 (−1.59, −0.08)	−1.09 (−3.17, 0.99)	−2.49 (−5.30, 0.32)	−0.62 (−1.29, 0.05)

* Adjusted with race, employment, marital status, insurance, menopausal status, tobacco use, and alcohol use.

**Table 4:** Association between ADI score and built environment variables and overall survival among breast cancer patients.

	Model 1	Model 2	Model 3	Model 4	Model 5	Model 6
	HR (95% CI)	HR (95% CI)	HR (95% CI)	HR (95% CI)	HR (95% CI)	HR (95% CI)
ADI	1.06 (1.02, 1.10)	1.04 (1.01, 1.09)	1.02 (0.98, 1.06)	1.02 (0.98, 1.06)	1.03 (0.98, 1.08)	0.98 (0.94, 1.03)
PM2.5	0.92 (0.83, 1.03)	0.90 (0.80, 0.99)	0.78 (0.70, 0.88)	0.88 (0.79, 0.98)	0.85 (0.75, 0.96)	0.71 (0.63, 0.82)
NDVI	1.45 (0.39, 5.33)	1.69 (0.45, 6.34)	2.58 (0.68, 9.83)	1.65 (0.43, 6.28)	1.67 (0.36, 7.81)	2.79 (0.58, 13.33)
mRFEI						
Low (=0)	Reference	Reference	Reference	Reference	Reference	Reference
High (>0)	0.82 (0.65, 1.03)	0.80 (0.64, 1.01)	0.82 (0.65, 1.03)	0.80 (0.64, 1.01)	0.81 (0.62, 1.05)	0.79 (0.61, 1.03)
RFAI						
Low (=0)	Reference	Reference	Reference	Reference	Reference	Reference
High (>0)	0.91 (0.76, 1.09)	0.90 (0.75, 1.08)	0.91 (0.76, 1.09)	0.89 (0.74, 1.06)	0.96 (0.78, 1.19)	0.92 (0.74, 1.14)

Model 1: crude. Model 2: adjusted demographic variables, including age, race, and menopausal status. Model 3: adjusted demographic variables and individual SES factors, including employment, marital status, and insurance type. Model 4: adjusted demographic variables and lifestyle factors, including alcohol use and tobacco use. Model 5: adjusted demographic variables and clinical variables, including tumor stage, ER status, TNBC status, and treatment. Model 6: adjusted demographic, individual SES, lifestyle, and clinical factors.

**Table 5:** Associations between ADI and built environment variables and overall survival by tumor subtypes.

	ER+ (N = 1950)	ER− (N = 361)	TNBC (N = 211)	Non-TNBC (N = 2466)
	HR (95% CI) *	HR (95% CI) *	HR (95% CI) *	HR (95% CI) *
ADI	0.99 (0.94, 1.05)	0.99 (0.89, 1.10)	0.93 (0.82, 1.06)	1.01 (0.96, 1.06)
PM2.5	0.72 (0.63, 0.82)	0.62 (0.47, 0.82)	0.57 (0.39, 0.83)	0.71 (0.63, 0.80)
NDVI	1.89 (0.32, 11.19)	20.84 (0.64, 682.38)	6.60 (0.10, 458.20)	3.56 (0.69, 18.64)
mRFEI				
Low (=0)	Reference	Reference	Reference	Reference
High (>0)	0.84 (0.62, 1.12)	0.45 (0.23, 0.85)	0.40 (0.18, 0.90)	0.85 (0.65, 1.10)
RFAI				
Low (=0)	Reference	Reference	Reference	Reference
High (>0)	1.01 (0.79, 1.28)	0.61 (0.38, 0.97)	0.48 (0.26, 0.87)	1.06 (0.86, 1.32)

* Adjusted for age, race, employment, marital status, insurance, menopausal status, tobacco use, alcohol use, stage, and treatment.

## Data Availability

All data generated or analyzed during this study are included in this published article and its Supplementary Information files.

## Supplementary Materials

The following supporting information can be downloaded at: https://www.mdpi.com/article/10.3390/cancers17091502/s1, Table S1: Associations of neighborhood variables and tumor characteristics: sensitive analysis. Table S2: Association between PM2.5 and overall survival.

## References

[1] Arnold M., Morgan E., Rumgay H., Mafra A., Singh D., Laversanne M., Vignat J., Gralow J.R., Cardoso F., Siesling S. (2022). Current and future burden of breast cancer: Global statistics for 2020 and 2040. Breast.

[2] Minas T.Z., Kiely M., Ajao A., Ambs S. (2021). An overview of cancer health disparities: New approaches and insights and why they matter. Carcinogenesis.

[3] Hill H.E., Schiemann W.P., Varadan V. (2020). Understanding breast cancer disparities—A multi-scale challenge. Ann. Transl. Med..

[4] Obeagu E.I., Obeagu G.U. (2024). Breast cancer: A review of risk factors and diagnosis. Medicine.

[5] Gomez S.L., Shariff-Marco S., DeRouen M., Keegan T.H., Yen I.H., Mujahid M., Satariano W.A., Glaser S.L. (2015). The impact of neighborhood social and built environment factors across the cancer continuum: Current research, methodological considerations, and future directions. Cancer.

[6] Fuemmeler B.F., Shen J., Zhao H., Winn R. (2023). Neighborhood deprivation, racial segregation and associations with cancer risk and outcomes across the cancER-control continuum. Mol. Psychiatry.

[7] Chen N., Mita C., Chowdhury-Paulino I.M., Shreves A.H., Hu C.R., Yi L., James P. (2024). The built environment and cancer survivorship: A scoping review. Health Place.

[8] Wray A.J.D., Minaker L.M. (2019). Is cancer prevention influenced by the built environment? A multidisciplinary scoping review. Cancer.

[9] Aoki R.F., Uong S.P., Gomez S.L., Alexeeff S.E., Caan B.J., Kushi L.H., Torres J.M., Guan A., Canchola A.J., Morey B.N. (2021). Individual- and neighborhood-level socioeconomic status and risk of aggressive breast cancer subtypes in a pooled cohort of women from Kaiser Permanente Northern California. Cancer.

[10] Chen J.C., Handley D., Elsaid M.I., Fisher J.L., Plascak J.J., Anderson L., Tsung C., Beane J., Pawlik T.M., Obeng-Gyasi S. (2024). Persistent Neighborhood Poverty and Breast Cancer Outcomes. JAMA Netw. Open.

[11] Bhattacharyya O., Li Y., Fisher J.L., Tsung A., Eskander M.F., Hamad A., Obeng-Gyasi S. (2021). Low neighborhood socioeconomic status is associated with higher mortality and increased surgery utilization among metastatic breast cancer patients. Breast.

[12] Goel N., Hernandez A.E., Mazul A. (2024). Neighborhood Disadvantage and Breast CancER-Specific Survival in the US. JAMA Netw. Open.

[13] Goel N., Hernandez A., Thompson C., Choi S., Westrick A., Stoler J., Antoni M.H., Rojas K., Kesmodel S., Figueroa M.E. (2023). Neighborhood Disadvantage and Breast CancER-Specific Survival. JAMA Netw. Open.

[14] Roy A.M., George A., Attwood K., Alaklabi S., Patel A., Omilian A.R., Yao S., Gandhi S. (2023). Effect of neighborhood deprivation index on breast cancer survival in the United States. Breast Cancer Res. Treat..

[15] Barber L.E., Maliniak M.L., Moubadder L., Johnson D.A., MillER-Kleinhenz J.M., Switchenko J.M., Ward K.C., McCullough L.E. (2024). Neighborhood Deprivation and Breast Cancer Mortality Among Black and White Women. JAMA Netw. Open.

[16] Coughlin S.S., Smith S.A. (2015). The Impact of the Natural, Social, Built, and Policy Environments on Breast Cancer. J. Environ. Health Sci..

[17] Obeng-Gyasi S., Obeng-Gyasi B., Tarver W. (2022). Breast Cancer Disparities and the Impact of Geography. Surg. Oncol. Clin. N. Am..

[18] Cannioto R.A., Attwood K.M., Davis E.W., Mendicino L.A., Hutson A., Zirpoli G.R., Tang L., Nair N.M., Barlow W., Hershman D.L. (2023). Adherence to Cancer Prevention Lifestyle Recommendations Before, During, and 2 Years After Treatment for High-risk Breast Cancer. JAMA Netw. Open.

[19] White A.J., Fisher J.A., Sweeney M.R., Freedman N.D., Kaufman J.D., Silverman D.T., Jones R.R. (2024). Ambient fine particulate matter and breast cancer incidence in a large prospective US cohort. J. Natl. Cancer Inst..

[20] Eve L., Fervers B., Le Romancer M., Etienne-Selloum N. (2020). Exposure to Endocrine Disrupting Chemicals and Risk of Breast Cancer. Int. J. Mol. Sci..

[21] Guo Q., Wang X., Gao Y., Zhou J., Huang C., Zhang Z., Chu H. (2021). Relationship between particulate matter exposure and female breast cancer incidence and mortality: A systematic review and meta-analysis. Int. Arch. Occup. Environ. Health.

[22] Zhang Z., Yan W., Chen Q., Zhou N., Xu Y. (2019). The relationship between exposure to particulate matter and breast cancer incidence and mortality: A meta-analysis. Medicine.

[23] Kind A.J.H., Buckingham W.R. (2018). Making Neighborhood-Disadvantage Metrics Accessible—The Neighborhood Atlas. N. Engl. J. Med..

[24] van Donkelaar A., Martin R.V., Li C., Burnett R.T. (2019). Regional Estimates of Chemical Composition of Fine Particulate Matter Using a Combined Geoscience-Statistical Method with Information from Satellites, Models, and Monitors. Environ. Sci. Technol..

[25] Xu R., Huang X., Zhang K., Lyu W., Ghosh D., Li Z., Chen X. (2023). Integrating human activity into food environments can better predict cardiometabolic diseases in the United States. Nat. Commun..

[26] Kim H.J., Kim S., Freedman R.A., Partridge A.H. (2022). The impact of young age at diagnosis (age <40 years) on prognosis varies by breast cancer subtype: A U.S. SEER database analysis. Breast.

[27] Tagliabue G., Borgini A., Tittarelli A., van Donkelaar A., Martin R.V., Bertoldi M., Fabiano S., Maghini A., Codazzi T., Scaburri A. (2016). Atmospheric fine particulate matter and breast cancer mortality: A population-based cohort study. BMJ Open.

[28] Mokbel K. (2024). Breath of Danger: Unveiling PM2.5’s Stealthy Impact on Cancer Risks. Anticancer Res..

[29] Hu H., Dailey A.B., Kan H., Xu X. (2013). The effect of atmospheric particulate matter on survival of breast cancer among US females. Breast Cancer Res. Treat..

[30] Prada D., Baccarelli A.A., Terry M.B., Valdez L., Cabrera P., Just A., Kloog I., Caro H., Garcia-Cuellar C., Sanchez-Perez Y. (2021). Long-term PM(2.5) exposure before diagnosis is associated with worse outcome in breast cancer. Breast Cancer Res. Treat..

[31] Sierra-Vargas M.P., Montero-Vargas J.M., Debray-Garcia Y., Vizuet-de-Rueda J.C., Loaeza-Roman A., Teran L.M. (2023). Oxidative Stress and Air Pollution: Its Impact on Chronic Respiratory Diseases. Int. J. Mol. Sci..

[32] Mahendra A., Polsky J.Y., Robitaille E., Lefebvre M., McBrien T., Minaker L.M. (2017). Status report—Geographic retail food environment measures for use in public health. Health Promot. Chronic Dis. Prev. Can..

[33] Cerceo E., Sharma E., Boguslavsky A., Rachoin J.S. (2023). Impact of Food Environments on Obesity Rates: A State-Level Analysis. J. Obes..

[34] Seiler A., Chen M.A., Brown R.L., Fagundes C.P. (2018). Obesity, Dietary Factors, Nutrition, and Breast Cancer Risk. Curr. Breast Cancer Rep..

[35] Braakhuis A.J., Campion P., Bishop K.S. (2016). Reducing Breast Cancer Recurrence: The Role of Dietary Polyphenolics. Nutrients.

[36] Burwell A., Kimbro S., Mulrooney T. (2023). Geospatial Associations between Female Breast Cancer Mortality Rates and Environmental Socioeconomic Indicators for North Carolina. Int. J. Environ. Res. Public Health.

[37] Bevel M.S., Tsai M.H., Parham A., Andrzejak S.E., Jones S., Moore J.X. (2023). Association of Food Deserts and Food Swamps With Obesity-Related Cancer Mortality in the US. JAMA Oncol..

