# Correction: Shen et al. Neighborhood Disadvantage, Built Environment, and Breast Cancer Outcomes: Disparities in Tumor Aggressiveness and Survival. *Cancers* 2025, *17*, 1502

**DOI:** 10.3390/cancers17132231

**Published:** 2025-07-03

**Authors:** Jie Shen, Yufan Guan, Supraja Gururaj, Kai Zhang, Qian Song, Xin Liu, Harry D. Bear, Bernard F. Fuemmeler, Roger T. Anderson, Hua Zhao

**Affiliations:** 1 Department of Public Health Sciences, School of Medicine, University of Virginia, Charlottesville, VA 22903, USA; cua9zd@virginia.edu (J.S.); uyt8pf@virginia.edu (Y.G.); supraja.guruarj@virginia.ed (S.G.); ra2ee@virginia.edu (R.T.A.); 2 Department of Population and Community Health, College of Public Health, The University of North Texas Health Science Center at Fort Worth, Fort Worth, TX 76107, USA; zhang@umich.edu; 3 Department of Gerontology, Donna M and Robert J Manning College of Nursing and Health Sciences, University of Massachusetts, Boston, MA 02125, USA; song@umass.edu; 4 School of Public Health and Emergency Management, Southern University of Science and Technology, Shenzhen 518055, China; xin@sust.edu.cn; 5 Departments of Surgery, School of Medicine, Virginia Commonwealth University, Richmond, VA 23284, USA; harry@vcu.edu; 6 Departments of Family Medicine, School of Medicine, Virginia Commonwealth University, Richmond, VA 23284, USA; fuemmeler@vcu.edu

In the published publication [1], the Acknowledgements Section is missing. The authors apologize for any inconvenience caused and state that the scientific conclusions are unaffected. This correction was approved by the Academic Editor. The original publication has also been updated. In this correction, we would like to include the following:

**Acknowledgments:**  We wish to thank The Population Health and Cancer Outcomes Core at the University of Virginia Comprehensive Cancer Center for providing research support on electronic health data extraction and IRB assistance.
