# Supplementary material for: Neighborhood Disadvantage, Built Environment, and Breast Cancer Outcomes: Disparities in Tumor Aggressiveness and Survival
Source: Cancers (Basel). 2025 Apr 29;17(9):1502. doi: 10.3390/cancers17091502 (PMC12070865; doi:10.3390/cancers17091502)
Supplement: Supplementary file 1 [file cancers-17-01502-s001.zip › cancers-3562774-supplementary.pdf]

Supplement Tables

Supplement Table S1. Associations of neighborhood variables and tumor characteristics-sensitive analysis						
	Tumor stage		Tumor grade		ER status	TNBC status
	II vs I	III vs I	Moderately vs Well	Poorly vs Well	Negative vs Positive	Yes vs No
	OR(95%CI)	OR(95%CI)	OR(95%CI)	OR(95%CI)	OR(95%CI)	OR(95%CI)
PM2.5 (1 year)	1.09 (0.99,1.21)	**1.20 (1.04,1.39)**	-	-	-	-
PM2.5 (3 year)	**1.22 (1.11,1.34)**	**1.25 (1.10,1.43)**	-	-	-	-

1.Adjusted with age, race, employment, marital status, insurance, menopausal status, tobacco use, and alcohol use.

Supplement Table S2. Association between PM2.5 and overall survival

	Model1<br>HR (95%CI)	Model2<br>HR (95%CI)	Model3<br>HR (95%CI)	Model4<br>HR (95%CI)	Model5<br>HR (95%CI)	Model6<br>HR (95%CI)
PM2.5-1year	0.92(0.83,1.03)	**0.90(0.80,0.99)**	**0.78(0.70,0.88)**	**0.88(0.79,0.98)**	**0.85(0.75,0.96)**	**0.71(0.63,0.82)**
PM2.5-3year	0.92(0.83,1.01)	**0.89(0.80,0.98)**	**0.76(0.68,0.84)**	**0.86(0.76,0.95)**	**0.84(0.75,0.96)**	**0.69(0.61,0.78)**

1.Adjusted with age, race, employment, marital status, insurance, menopausal status, tobacco use, and alcohol use.
